# Knowledge and Attitude of Pharmacy Students toward People with Mental Illnesses and Help-Seeking: A Cross-Sectional Study from Saudi Arabia

**DOI:** 10.3390/pharmacy9020082

**Published:** 2021-04-16

**Authors:** Saud Alsahali

**Affiliations:** Department of Pharmacy Practice, Unaizah College of Pharmacy, Qassim University, Qassim 51911, Saudi Arabia; s.alsahali@qu.edu.sa; Tel.: +966-16-3011503

**Keywords:** mental illness, help-seeking, stigma, knowledge, attitude, mental disorders

## Abstract

People with mental illnesses (MIs) face several challenges in addition to their disease. People’s negative views of those with MIs impact patients’ decisions to seek professional help. The aims of this study were to assess pharmacy students’ attitudes toward people with MIs and seeking help for mental health, as well as their knowledge about the causes of MIs. A cross-sectional survey was conducted on pharmacy students at Unaizah College of Pharmacy, Qassim University, Saudi Arabia. Out of the 460 distributed questionnaires, 330 complete questionnaires were received, giving a response rate of 71.7%. Overall, the mean total score for attitude towards people with MIs was 60.16 ± 10.48 (maximum attainable score: 105). In this study, 51.12% believed that people with MIs are more likely to harm others than a person without MIs and 66.9% mentioned that they did not trust the work of a mentally ill person as part of their work team. However, only 35.45% believed that it is difficult for mentally ill individuals to follow social rules. In terms of attitudes toward help-seeking, the mean total score of was 12.83 ± 3.16 out of the maximum score of 25. In addition, the mean total score for knowledge about causes of mental illness was 2.92 ± 1.76 out of the maximum score of 8. The participants reported that MIs could be due to genetic inheritance (56%), substance abuse (54.5%), or brain disease (66.1%). The findings showed that there are some negative attitudes toward people with MIs and negative attitudes towards seeking help for mental health. In addition, some misconceptions about the causes of MIs are prevalent. Consequently, the incorporation of more topics concerning mental health in pharmacy curricula could help improve the awareness of and knowledge about mental health.

## 1. Introduction

People with mental illnesses (MIs) often face several challenges in carrying out daily activities [1]. Many of them experience discrimination and difficulties in finding suitable employment, as well as a lack of access to health care and education facilities [2,3]. Most of these challenges stem from misunderstood concepts of mental diseases, including negative views and stereotypes, which are prevalent in many societies [1]. In addition, stigma toward MIs is prevalent in many societies. This includes fears, negative attitudes toward patients with MIs, and avoidance of dealing with them on a regular basis. This stigma remains attached to many societies despite improvement in treating patients with mental disorders [3,4]. In 2017, the estimated prevalence of MI in the Unites States was 46 million, which represents approximately 20% of the adult population [5]. A meta-analysis study published in 2014, which included 155 surveys conducted in different communities across 59 countries, showed that about one in five persons had experienced a mental disorder within a period of twelve months [6].

Despite the improvement in treatment, many individuals do not seek healthcare due to stigma, which may lead to deterioration of their health conditions and result in negative occupational outcomes and isolation from the community, thus leading to hindrances in their social life [7]. As a result of these negative views, those with MIs might lose out on opportunities to improve their quality of life, face inequalities in the competition to secure employment, and experience difficulties in acquiring good housing and access to good health care [1].

Many studies showed that help-seeking intentions were associated with a positive attitude and better knowledge combined with community support [8,9]. Thus, providing reliable information about MIs will be helpful in reducing negative attitudes about mental illness within the society. Moreover, several studies suggest that attending awareness programs about MIs will help improve attitudes toward persons with mental disorders [10,11]. These programs have been proven to be effective across different communities, including adolescents, college students, and persons with mental disorders [1].

Several tools for assessing the attitudes of people toward those with MIs were developed to exhibit the views among different cultures and community groups [12,13]. It was reported that some people feel that MI patients are dangerous, incurable, and untrustworthy and considered a diagnosis with an MI to be shameful [13]. Patients with MI who seek early professional help could benefit more than those who delay seeking help. More awareness about diseases and treatment guides will help people with MIs to seek professional help. However, people have varied views toward seeking help from mental health centers [14].

There is growing concern about attitudes toward people with MIs and their effect on their treatment and social lives [1]. The levels of knowledge and attitudes of students at health colleges including pharmacy students towards MIs are of paramount importance, as they will be future health care professionals. The awareness and knowledge of these future healthcare professionals about MIs will help the community to better deal with negative attitudes toward MIs. Consequently, the aims of the study were to assess the attitudes toward people with MIs, attitudes toward seeking help for mental health, and the level of knowledge about causes of MIs among students at Unaizah College of Pharmacy, Qassim University, Saudi Arabia.

## 2. Materials and Methods

### 2.1. Study Design and Setting

This was a cross-sectional questionnaire-based study. The target population of this study was pharmacy students at Unaizah College of Pharmacy, Qassim University, Saudi Arabia. Consequently, all 460 students enrolled in the Doctor of Pharmacy (PharmD) program at the college were invited to take part in the study.

### 2.2. Development of the Questionnaire

The questionnaire used in this study consisted of three parts, which covered attitudes toward MIs, attitudes toward help-seeking, and the level of knowledge about the causes of MIs. The first part was adapted from a previous questionnaire that measured the attitudes toward MIs across communities and cultures [13]. It consisted of 21 statements that represented common negative views toward patients with mental illness. The 21 statements focused on three factors and views toward mental illness, which were dangerousness, poor interpersonal and social skills, and incurability. The statements were rated on a six-point Likert scale ranging from “completely disagree” (0) to “completely agree”(5). The range of possible total scores was from 0 to 105, in which a higher score reflected a high level of negative views toward people with MIs. To summarize the results in a simple way, the percentage of participants who agreed/completely agreed were combined and reported for each statement.

The second part of the questionnaire assessed the attitudes toward help-seeking, and this was informed by previous studies conducted in Saudi Arabia and Europe [14,15]. This part consisted of five hypothetical statements and the answers were measured on a five-point Likert-scale ranging from “strongly agree”, which was equal to five points, to “strongly disagree”, which was equal to one point. For the negative statements, the score of the Likert scale was reversed and graded using “strongly disagree” with five points and “strongly agree” with one point. The attainable score ranged from 5 to 25 points. For this scale, a higher score reflected a high level of positive attitudes toward help-seeking behavior.

The third part of the questionnaire assessed participants’ knowledge about the causes of MIs. It consisted of eight questions about the causes of mental illness, which were reported previously in the literature [14]. The participants received one point if their answer was correct and received zero if their answer was wrong or if they did not know the answer. The attainable score ranged from 0 to 8 points.

### 2.3. Administration of the Survey

The questionnaire was distributed by trained data collectors who approached the students and invited them to take part in the study. The participants were provided with a brief overview of the study, including its aim and the fact that participation was totally voluntary and that they could withdraw at any time during the study. Once the questionnaires were completed, they were collected and returned back to the principal investigator. The study was conducted from December 2019 to January 2020.

### 2.4. Analysis of the Results

SPSS version 20.0 (IBM Corp., Armonk, NY, USA) was used to analyze the data and to summarize participants’ responses. Descriptive statistics (i.e., frequencies and percentages) were used to summarize the students’ responses to the questionnaire questions and statements. Inferential statistics (i.e., Student’s *t*-test) were used to examine whether there were differences in the means of the scores of the variables. A *p*-value of <0.05 was considered statistically significant.

## 3. Results

### 3.1. Response Rate and Demographic Data

Out of the460 distributed questionnaires, 372 responses were received. Among these 372 returned questionnaires,42 responses were incomplete and were excluded. Consequently, 330 valid questionnaires were used in this study, giving a response rate of 71.7%. The participants comprised 133 men (40.3%) and 197 women (59.7%). The age range of the participants was between 19 and 25 years. A total of 48 participants (14.54%) had a family member with mental illness, as illustrated in Table 1

### 3.2. Participants’ Attitudes toward People with MI

Overall, the mean total score for attitudes towards people with MI was 60.16 ± 10.48. In this study, it was found that two-thirds of the participants (66.9%) did not trust work done by mentally ill individuals. Moreover, 67.6% of the participants believed that people with psychological illnesses displayed unpredictable behavior. Fifty-nine percent of the respondents believed that people with MIs tend to be criminals. Additionally, 78.8% of the participants believed that when people with MIs received treatment one time, they were more likely to need further treatment in the future. It was found that more than a half of the participants (55.8%) believed that people with MIs cannot live independently since they are unable to assume responsibilities, and 51.8% of participants suggested that people with a mental disorder should have a job with only minor responsibilities, as shown in Table 2. In this study, there was no statistically significant difference in the mean score of attitude towards people with MIs between male participants (mean (SD) = 61.08 ± 11.9) and female participants (mean (SD) = 59.56 ± 10.3) (*p* = 0.197)

### 3.3. Attitudes toward Help-Seeking

Overall, the mean total score for attitudes toward help-seeking was 12.83 ± 3.16. In this study, as shown in Table 3, it was found that more than one-third (36%) of the participants agreed that people with MIs would seek professional help at mental health centers, and 32.4% of them would visit health care providers if they faced emotional problems. Sixty percent of participants disagreed that they would feel comfortable if they talked about their personal problems with health care providers, and 48.8% of them reported that they would be embarrassed if their friends knew about them receiving professional help for emotional issues. As shown in this part, there was a statistically significant difference in the mean score for attitudes toward help-seeking between male participants (mean (SD) = 13.56 ± 3.45) and female participants (mean (SD) = 12.35 ± 2.86) (*p* = 0.001).

### 3.4. Knowledge about the Causes of Mental Illness

Overall, the mean total score for knowledge about the causes of mental illnesses was 2.92 ± 1.76. In addition, as shown in Table 4, regarding the causes of mental illnesses,56%of the participants agreed that they are caused by genetic inheritance, 54.5% agreed that they are caused by substance abuse, and 79.4% believed that they are caused by bad things happening. Approximately two-thirds of the participants (66.1%) reported that mental illnesses were caused by brain disease, 30% believed that they were caused by personal weakness, and 27.9% saw them as a result of poverty. The female participants (mean (SD) = 3.21 ± 1.78) had significantly better knowledge about the causes of mental illnesses compared to the male participants (mean (SD) = 2.49 ± 1.65) (*p* <001).

## 4. Discussion

Several recent studies clearly indicated that there is a widespread negative attitude toward people with mental illnesses. Negative views exist in many communities including students, the general population, and workers in health care [12,14,16,17,18]. The results of this study were comparable to those of several previous studies that were conducted to assess the attitudes toward mental illness among students [12,16]. Approximately 59% of the students in our sample believed that people with mental disorderswere more likely to be criminally predisposed. This is similar to the results reported from India in which 54% of students agreed that people with mental illness have a greater tendency to be criminals [16]. In the literature, it was reported that students tended to label the people who experience symptoms of mental illnesses as dangerous people and were less likely to interact with them [19].

The misconception about individuals with mental health problems could lead to increased social disconnection, especially with people with psychotic disorders [20]. A total of 67.6% of the participants believed that the behaviors of those with mental illnesses are unpredictable. A survey conducted in Germany showed that 49.6% of the general public believed that the behavior of a person with mental illness was unpredictable [21]. Moreover, it was found that the general public believed that people with mental illnesses are more likely to engage in criminal and violent acts more than members of the general public [4,12,22,23,24]

In the present study, more than two-thirds of the participants did not trust the work of a person with a mental illness, and 55.8% believed that they cannot live independently as they cannot assume responsibilities. Although many individuals with mental illnesses are willing to work, they often face discrimination from the general population [25]. In 2020, a Saudi study conducted among young adults showed that 40% of participants had a moderately negative attitude toward the ability of people with mental illness to make friends, to be parents, or to be a trustworthy team member [26]. Ina study conducted in the UK, it was found that people with mental disorders are up to 40% less likely to find a job compared to other disability groups [27]. These findings may illustrate that people with mental illnesses face difficulties in finding jobs, which leads to an increased unemployment rate among those with MIs.

In 2015, a study conducted in Hong Kong showed that only 26% of people with mental disorders had been to doctors for their diseases [28]. Another study conducted in China, with a sample size of more than 63,000 adults, showed that 24.0% of the respondents of the sample had a mental disorder; however, only 8.0% of them sought professional help [29]. In this study, only 36% of participants agreed that they would seek professional help if they needed it. This percentage was less than that obtained in a study conducted by Aboalfotouh et al. (2019) among the Saudi public, which showed that 45% of the participants agreed that people with mental disorders would seek professional help at mental health centers [14]. Many researchers concluded that a negative attitude toward mental illnesses and seeking professional help was the main barrier in the path to achieving an overall healthy and active life [30,31]. Additionally, they found that the fear of discrimination and the family’s resistance to help-seeking may act as a catalyst in increasing this negative attitude in the community [32].

Although the exact causes of mental disorders are not yet well-known, there are many risk factors, such as biological, environmental, and social factors that may increase the chance of having mental disorders. Gathering accurate information is considered as an effective way to reduce negative views amongst the general public [32]. Many studies have proven that poor knowledge of mental disorders increases negative views against people with mental illnesses [33,34,35]. In our study, inadequate knowledge amongst the participants was observed, with women having better knowledge than men. The low level of knowledge about mental illnesses in this study is comparable with the findings of the study conducted by Aboalfotouh et al. (2019) in Saudi Arabia, in which 87.9% of the Saudi public had inadequate knowledge about this issue [14]. The complications of untreated cases may affect the health care system in the long run. Children with MIs may face difficulties in learning at school, and adolescents may quit school and become less effective members of society [35]. In addition, they may add financial pressure on the health care system. In 2013, the US health care system spent more than 187 billion dollars on mental health with an annual rate of increase of 3.7%, according to a study conducted by Dieleman et al. [36]. The healthcare organizations that support mental illnesses have important roles in decreasing these consequences. They could help by providing accurate information about mental disorders and stopping the spread of false information by the media. Furthermore, such organizations help to guide patients and their families to better understand the diseases and find ways for diagnosis and treatment, thus promoting seeking professional help at specialized centers [35].

## 5. Limitations

This study is cross-sectional in nature; therefore, the findings do not prove the causality and the parameters obtained at this specific time. This study was conducted among pharmacy students with a good response rate, which was about (71%), and the results were comparable to the results of other studies; however, the sample was unrepresentative of the entire population.

## 6. Conclusions

Despite advances in the social and psychological treatment of mental disorders, individuals with MIs still have difficulties in their treatment, which are mainly due to community barriers. These barriers include negative attitudes toward people with MIs, which negatively impact their decision to seek help in specialized centers. The consequences of patients remaining untreated could impact the social lives of patients and their chances to improve their quality of life. Furthermore, this adds a substantial cost on the health care system of the country. The results of this study, in combination with those of several studies conducted in different countries among the general population and among students, showed that misconceptions and negative views toward those with MIs are prevalent. More research is needed in order to address the consequences. Public health campaigns could help to increase the awareness of mental illnesses, to improve the attitudes of people, and to guide the patients’ families to seek help. For college students, adding mental health-related content to their courses may help to improve their knowledge about mental disorders and guide patients and their families to seek help at specialized centers.

## Figures and Tables

**Table 1:** Participants’ demographics information.

Variables	*n* = 330	%
Gender Male Female	133 197	40.3 59.7
Age ≥20 <20	279 51	84.54 15.45
Patient in Family Yes No	48 282	14.54 85.46

**Table 2:** Students’ responses to statements on attitudes toward people with mental disorders.

Statement	Agree/Completely Agree
	*n* (%)
1. A mentally ill person is more likely to harm others than a person without mental illness	169 (51.12)
2. Mental disorders would require a much longer period of time to be cured than other general diseases	169 (51.2)
3. It may be a good idea to stay away from people who have psychological disorders because their behavior is dangerous	172 (52.12)
4. The term “psychological disorder” makes me feel embarrassed	172 (52.12)
5. A person with a psychological disorder should have a job with only minor responsibilities	171 (51.81)
6. Mentally ill individuals are more likely to be criminals	195 (59)
7. Psychological disorder is recurrent	185 (56)
8. I am afraid of what my boss, friends, and others would think if I were diagnosed with a psychological disorder	144 (43.63)
9. Individuals diagnosed as mentally ill suffer from its symptoms throughout their life	144 (43.63)
10. People who have received psychological treatment once are likely to need further treatment	260 (78.78)
11. It might be difficult for mentally ill individuals to follow social rules such as being punctual or keeping promises	117 (35.45)
12. I would be embarrassed if people knew that I dated a person who once received psychological treatment	262 (79.33)
13. I am afraid of people who are suffering from psychological disorder because they may harm me	160 (48)
14. A person with psychological disorder is less likely to function well as a parent	191 (56.87)
15. I would be embarrassed if a person in my family became mentally ill	102 (30.9)
16. I believe that psychological disorder can never be completely cured	93 (28.18)
17. Mentally ill individuals are unlikely to be able to live by themselves because they are unable to assume responsibilities	184 (55.8)
18. Most people would not knowingly be friends with a mentally ill person	128 (38.7)
19. The behavior of people who have psychological disorders is unpredictable	223 (67.6)
20. Psychological disorder is unlikely to be cured regardless of treatment	188 (57)
21. I would not trust the work of a mentally ill person assigned to my work team	221 (66.9)

**Table 3:** Students’ responses to statements on attitudes toward seeking professional help.

Statement	SA	A	NS	D	SD
	(%)	(%)	(%)	(%)	(%)
1. People with mental illness usually seek professional help at mental health services	13.9	22.1	40.9	18.3	4.8
2. I would seek professional help in the case of a serious emotional problem	14.8	17.6	27.5	32.5	7.6
3. I would feel comfortable talking about personal problems with a professional	5.8	12.4	21.8	37.9	22.1
4. I would be embarrassed if my friends knew I was getting professional help for an emotional problem	22.4	26.4	23.6	18.8	8.8
5. I would perceive professional help as effective	1.5	39.7	43.3	12.4	3.1

SA = strongly agree, A = agree, NS = not sure, D = disagree, SD = strongly disagree.

**Table 4:** Students’ responses toquestions on knowledge about causes of mental disorders.

Question	Yes	No	Do Not Know
	*n* (%)	*n* (%)	*n* (%)
1. Mental illness is caused by genetic inheritance	185 (56)	87 (26.4)	58 (17.6)
2. Mental illness is caused by substance abuse	180 (54.54)	84 (25.46)	66 (20)
3. Mental illness is caused by bad things happening to you	262 (79.4)	30 (9.1)	38 (11.5)
4. Mental illness is God’s punishment	18 (5.5)	209 (63.3)	103 (31.2)
5. Mental illness is caused by brain disease	218 (66.1)	44 (13.3)	67 (20.3)
6. Mental illness is caused by a personal weakness	100 (30.3)	161 (48.8)	69 (20.9)
7. Poverty can be the cause of mental illness	92 (27.9)	177 (53.6)	61 (18.5)
8. Mental illness is caused by spirits	37 (11.2)	163 (49.4)	130 (39.4)

## Data Availability

The data will be available from the corresponding author upon reasonable request.
